# Inguinoscrotal Bladder Herniation With Mery’s Sign: Individualized Timing and Primary Repair in the Setting of Suspected Urinary Infection

**DOI:** 10.7759/cureus.115526

**Published:** 2026-08-31

**Authors:** Mauro A Cardenas, Jazmin Sáenz López, Paola I Cárdenas Rosales, Sergio Miguel Suárez Fuentes, Melisa Licon Meza, Akemy H Tsuyi Loya, Juan Jimenez, Alejandra M Sanchez Poumian, Miguel A Gonzalez Galaviz, Alejandra Zermeño, Ivan Lopez

**Affiliations:** 1 Surgery, Hospital General “Presidente Lázaro Cárdenas del Río”, Instituto de Seguridad y Servicios Sociales de los Trabajadores del Estado (ISSSTE), Chihuahua, MEX; 2 General Medicine, Autonomous University of Chihuahua, Chihuahua, MEX; 3 Medical Education, Autonomous University of Chihuahua, Chihuahua, MEX; 4 General Surgery, Hospital Regional "Valentin Gomez Farías", Zapopan, MEX; 5 General Surgery, Autonomous University of Chihuahua, Chihuahua, MEX; 6 Surgery, Autonomous University of Chihuahua, Chihuahua, MEX; 7 General Medicine, Autonomous University of Durango, Chihuahua, MEX; 8 Medicine, Autonomous University of Chihuahua, Chihuahua, MEX; 9 Pediatrics, Universidad de Guadalajara, Guadalajara, MEX; 10 General Surgery, Hospital Civil de Guadalajara, Guadalajara, MEX

**Keywords:** hernia repair, inguinal bladder hernia, inguinal hernia, inguinoscrotal hernia, mery’s sign

## Abstract

Urinary bladder involvement in inguinal hernias is an uncommon condition, predominantly affecting older men and potentially causing urinary obstruction, infection, and renal dysfunction. We present the case of a 79-year-old man with a six-month history of progressive right inguinoscrotal enlargement, lower urinary tract symptoms, hematuria, and a positive Mery’s sign. Laboratory studies revealed renal dysfunction and urinary abnormalities in the setting of suspected urinary tract infection. Computed tomography demonstrated a right inguinoscrotal hernia containing the urinary bladder, with associated bladder wall thickening. Surgical repair was initially deferred to allow clinical optimization and initiation of antimicrobial therapy. Five days later, persistent fever, leukocytosis, worsening urinary findings, flank pain, and persistent systemic inflammatory response despite antimicrobial therapy prompted surgical intervention. The hernia was reduced through a lower midline infraumbilical approach, and primary tissue repair without prosthetic mesh was performed. After antimicrobial escalation, renal function recovered, inflammatory markers decreased, and the patient was discharged without surgical complications. At the two-month follow-up, no hernia recurrence was observed.

Bladder-containing inguinoscrotal hernias should be considered in elderly men presenting with inguinal or scrotal masses and lower urinary tract symptoms. Computed tomography is valuable for diagnosis and surgical planning. When urinary infection is suspected or inflammatory findings are present, individualized timing and technique of repair may be appropriate in selected patients according to clinical response and operative findings.

## Introduction

Urinary bladder involvement in inguinal hernias is an uncommon clinical entity, accounting for approximately 1-4% of inguinal hernias. It occurs predominantly in older men and has been associated with chronic bladder outlet obstruction and increased intra-abdominal pressure. Although many patients remain asymptomatic, bladder-containing inguinoscrotal hernias may present with lower urinary tract symptoms and can lead to urinary obstruction, urinary tract infection, renal dysfunction, and other serious complications [1,2].

Preoperative diagnosis remains challenging, as bladder involvement may not be recognized clinically and has historically been identified intraoperatively. Changes in urinary symptoms associated with variations in the size of the inguinoscrotal mass may provide an important clinical clue. Mery’s sign, characterized by two-stage urination with the need for manual compression of the herniated mass to facilitate bladder emptying, is considered suggestive of bladder involvement [1,3]. Computed tomography (CT) is particularly useful for confirming bladder herniation, defining its extent, and identifying associated urinary tract abnormalities [1,4].

Surgical management generally consists of reduction of the herniated bladder followed by repair of the inguinal defect. Bladder resection is reserved for selected circumstances, including bladder wall necrosis, a true diverticulum, a severely constricted hernia neck, or an associated bladder neoplasm [1,2]. The timing and technique of repair should be guided by the patient's clinical condition and operative findings, particularly when urinary or systemic inflammatory findings are present.

We present a case of chronic right inguinoscrotal bladder herniation in an elderly patient presenting with Mery’s sign, hematuria, renal dysfunction, and findings suggestive of urinary tract infection. Because preoperative diagnosis of bladder-containing inguinal hernias remains uncommon, recognition of bladder involvement allows for more informed surgical planning. This case illustrates the diagnostic and therapeutic challenges that may arise when a bladder-containing inguinoscrotal hernia is accompanied by progressive urinary and systemic inflammatory findings. It also highlights the importance of integrating clinical signs, cross-sectional imaging, and the patient’s clinical response when determining the timing and surgical approach to repair, emphasizing that management should be individualized according to the clinical condition and operative findings of each patient.

## Case presentation

A 79-year-old man presented to the emergency department in May 2026 with a six-month history of progressive right hemiscrotal enlargement and a medical history of systemic arterial hypertension, type 2 diabetes mellitus, obstructive sleep apnea, and chronic tobacco and alcohol use. He was evaluated in the Urology outpatient clinic and diagnosed with a right inguinoscrotal hernia. During the subsequent months, he developed lower urinary tract symptoms, including dysuria, urinary urgency, and mixed urinary incontinence. He also reported two-stage bladder emptying and the need to manually compress the inguinoscrotal mass to facilitate urinary drainage. These findings were consistent with Mery's sign and raised clinical suspicion of urinary bladder involvement in the hernia. One week before admission, his urinary symptoms worsened, prompting him to seek medical attention.

On physical examination, there was marked enlargement of the right hemiscrotum with a non-reducible inguinoscrotal mass. Compression of the scrotal swelling resulted in visible urinary drainage through the indwelling urinary catheter. The abdomen was soft and non-distended, without signs of an acute abdomen.

Initial laboratory evaluation revealed renal dysfunction, with a blood urea nitrogen level of 43.4 mg/dL and serum creatinine of 1.7 mg/dL. The white blood cell count was 10.6 × 10³/µL, with a neutrophil count of 5.56 × 10³/µL (Table 1). Urinalysis showed marked hematuria, glucosuria, and sparse bacteriuria, with negative nitrites and 1-3 leukocytes per high-power field (Table 2). These findings, together with the patient's urinary symptoms and clinical presentation, prompted initial antimicrobial therapy and close clinical monitoring.

**Table 1:** Initial laboratory findings at hospital admission Laboratory results obtained at the time of hospital admission, including complete blood count, renal function, glucose, and serum electrolytes. Patient values are presented alongside the corresponding reference ranges and units.

Laboratory test	Patient’s value	Reference range	Units
Hemoglobin	15	14 - 18	g/dL
Hematocrit	45.4	42 - 52	%
Platelets	264	150 - 400	×10³/µL
Leukocytes	10.6	4.5 - 10.5	×10³/µL
Neutrophils	5.56	2.1 - 6.1	×10³/µL
Glucose	96.7	70 - 110	mg/dL
Blood urea nitrogen	43.4	5 - 18	mg/dL
Creatinine	1.7	0.7 - 1.2	mg/dL
Sodium	139	136 - 145	mmol/L
Potassium	4.6	3.5 - 5.1	mmol/L
Chloride	104	98 - 107	mmol/L

**Table 2:** Initial urinalysis findings at hospital admission Urinalysis results obtained at the time of hospital admission, showing marked hematuria and glucosuria with minimal leukocyturia, negative nitrites, and sparse bacteriuria, without overt inflammatory urinary findings.

Urinalysis	Patient’s value	Reference range	Units	
Color	Straw-colored	Pale yellow to amber	N/A	
Appearance	Slightly cloudy	Clear - Transparent	N/A	
Density	1.02	1.005 - 1.030	N/A	
pH	6	4.5 - 8.0	N/A	
Protein	Negative	Negative	mg/dL	
Glucose	300	Negative	mg/dL	
Ketones	Negative	Negative	mg/dL	
Hemoglobin	250	Negative	Ery/uI	
Nitrites	Negative	Negative	N/A	
Leukocytes per high-power field	1 - 3	0 - 5	/HPF	
Erythrocytes per high-power field	Abundant	0 - 3	/HPF	
Bacterium	Sparse	Absent to rare	/HPF	

CT of the abdomen and pelvis demonstrated a right inguinoscrotal hernia containing the urinary bladder, with associated bladder wall thickening (Figure 1). The urinary catheter balloon was visualized at the margin of the hernia defect (Figure 2), and CT provided detailed preoperative assessment of the hernia anatomy (Figures 1-3).

**Figure 1:**
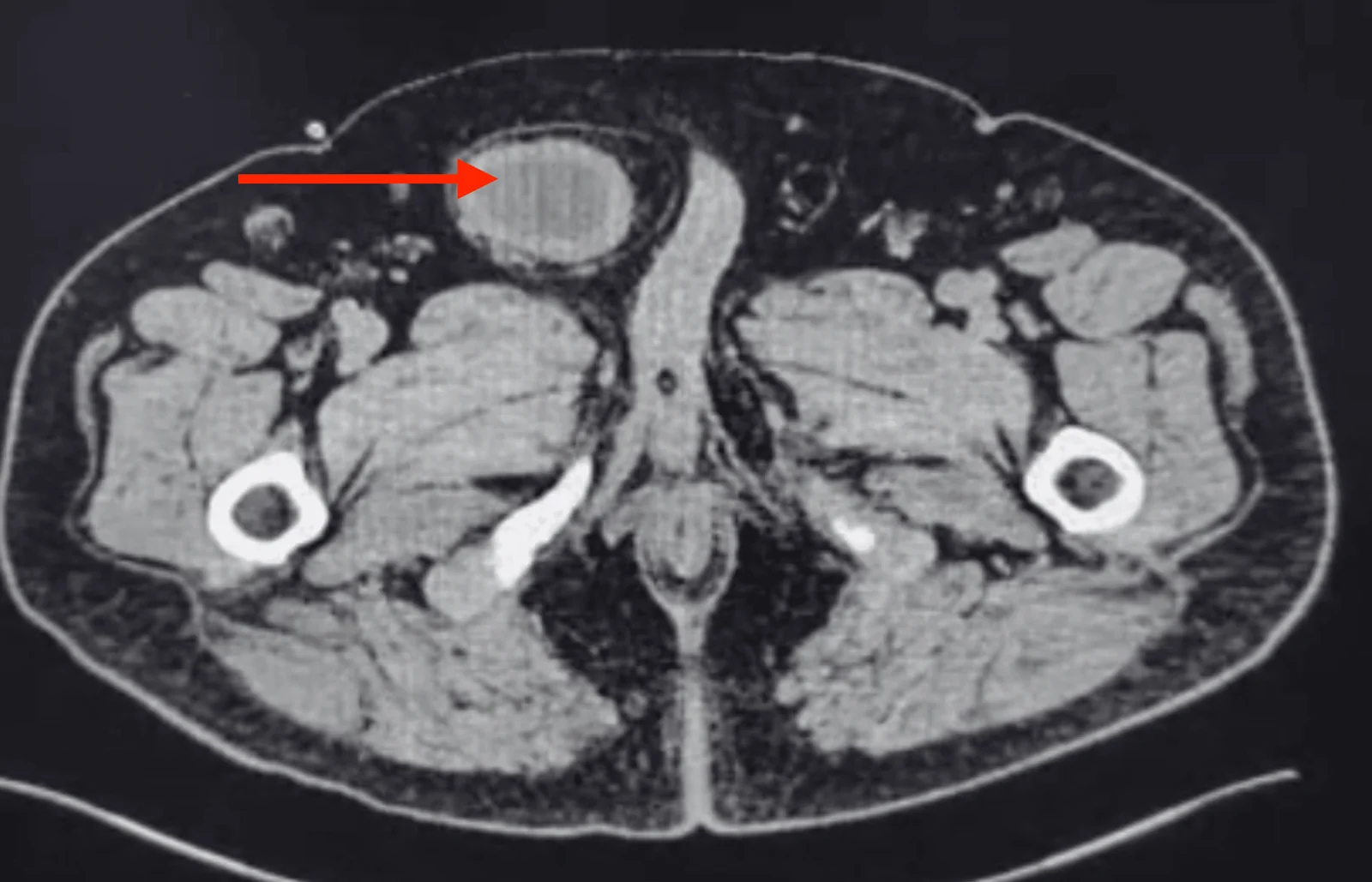
Axial computed tomography demonstrating the bladder-containing inguinoscrotal hernia Axial non-contrast computed tomography image demonstrating the urinary bladder herniating through the right inguinal canal, with associated bladder wall thickening indicated by the arrow.

**Figure 2:**
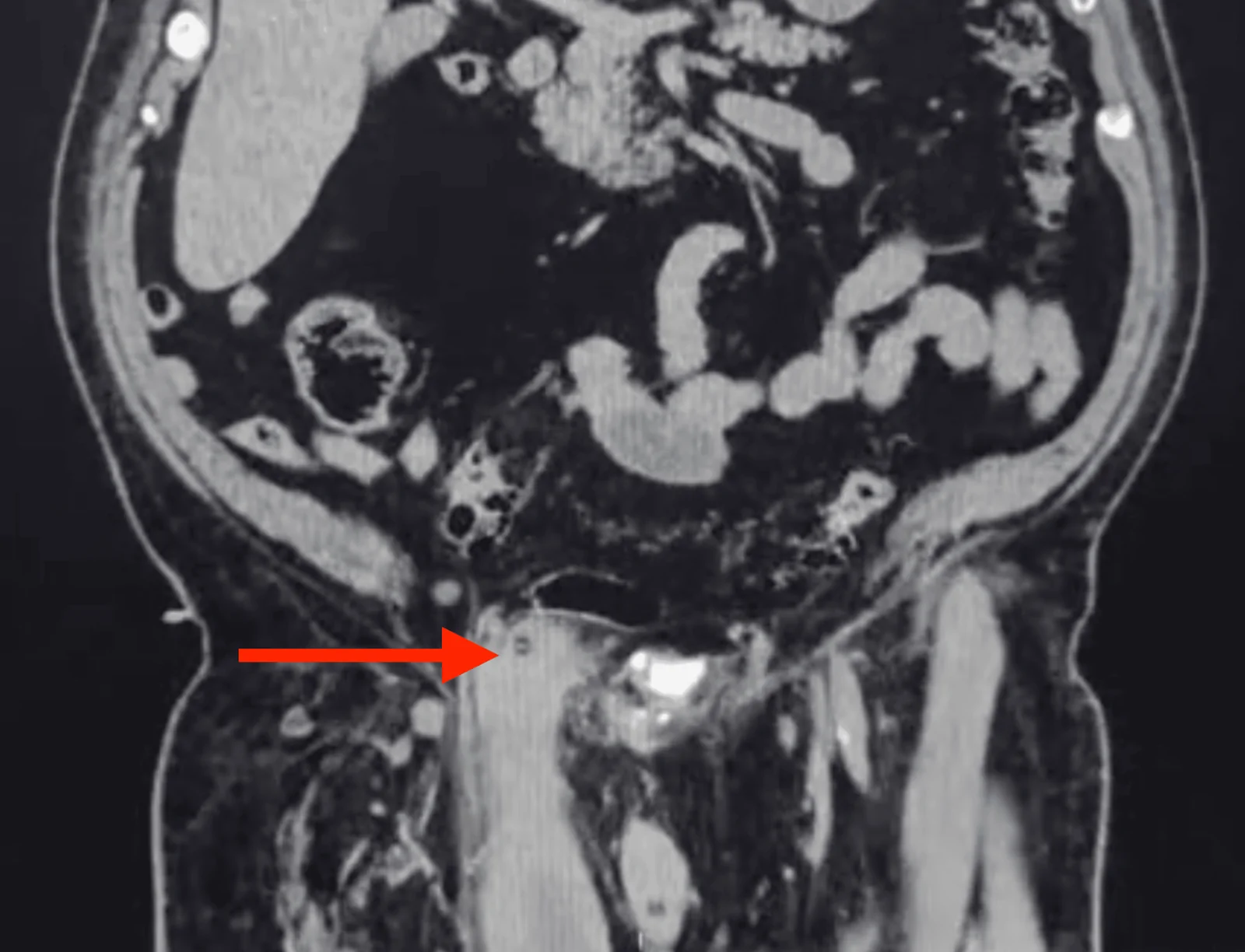
Coronal computed tomography showing the urinary catheter balloon at the hernia defect Coronal computed tomography image demonstrating the urinary bladder extending into the right inguinoscrotal hernia. The arrow indicates the urinary catheter balloon located at the edge of the hernia defect.

**Figure 3:**
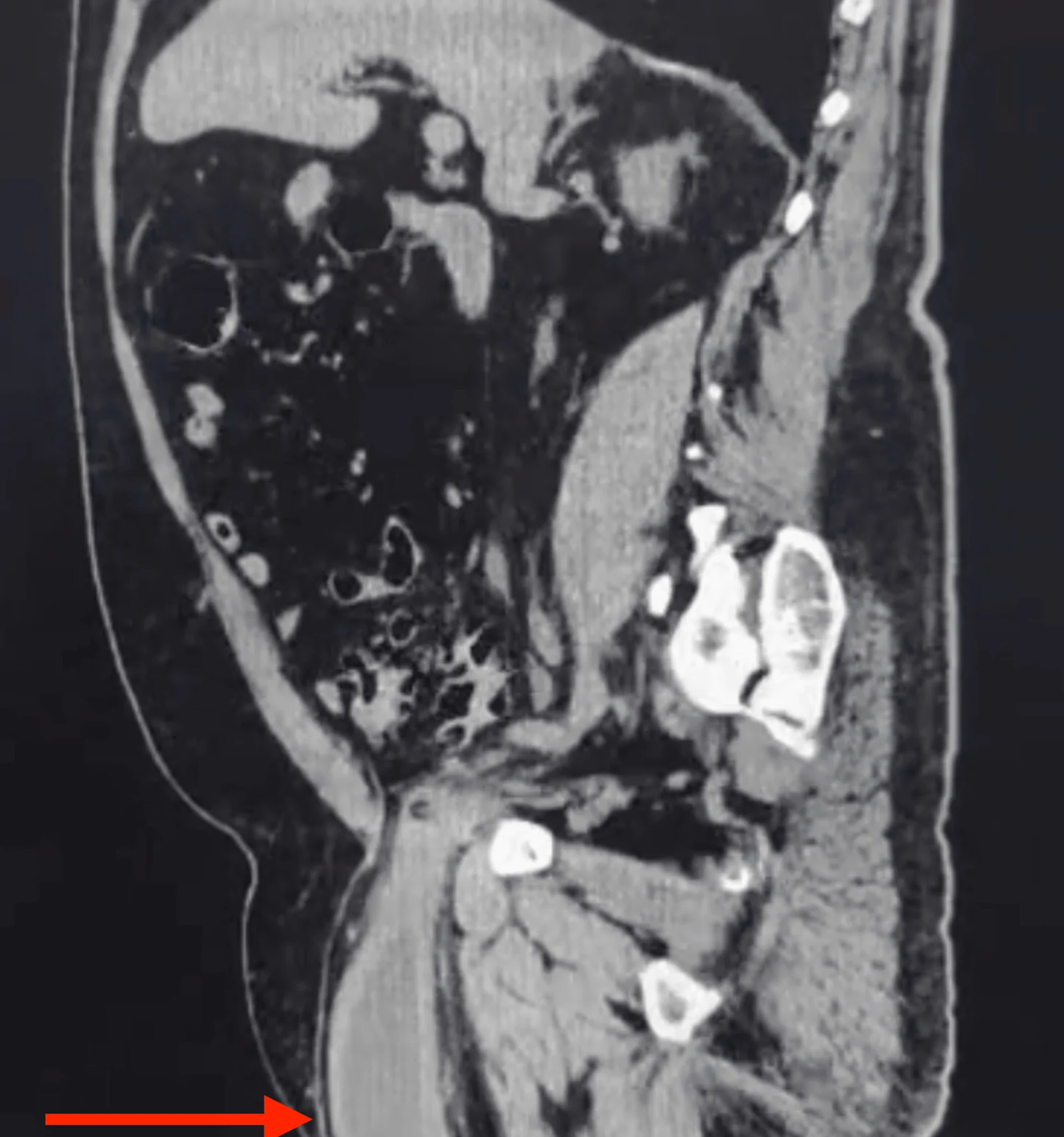
Sagittal computed tomography demonstrating bladder herniation into the right hemiscrotum Sagittal computed tomography image demonstrating extension of the urinary bladder into the right hemiscrotum, confirming the inguinoscrotal component of the hernia.

Because there was no immediate indication for emergent repair, definitive surgery was initially deferred to allow clinical optimization and assessment of the response to antimicrobial therapy. Five days after admission, the patient developed persistent fever, leukocytosis, neutrophilia, flank pain, and bilateral costovertebral angle tenderness. Repeat urinalysis demonstrated positive nitrites, abundant leukocytes, abundant bacteria, and persistent hematuria (Table 4). These findings represented progression of the urinary and systemic inflammatory picture despite antimicrobial therapy and prompted definitive surgical management.

**Table 3:** Laboratory findings five days after hospital admission Laboratory results obtained five days after hospital admission, demonstrating leukocytosis and neutrophilia with persistent elevation of blood urea nitrogen and serum creatinine. Patient values are presented alongside the corresponding reference ranges and units.

Laboratory test	Patient’s value	Reference range	Units
Hemoglobin	13.8	14 - 18	g/dL
Hematocrit	42.5	42 - 52	%
Platelets	249	150 - 400	×10³/µL
Leukocytes	17.5	4.5 - 10.5	×10³/µL
Neutrophils	14.5	2.1 - 6.1	×10³/µL
Glucose	104	70 - 110	mg/dL
Blood urea nitrogen	43.4	5 - 18	mg/dL
Creatinine	1.5	0.7 - 1.2	mg/dL
Sodium	145	136 - 145	mmol/L
Potassium	4.6	3.5 - 5.1	mmol/L
Chloride	112	98 - 107	mmol/L

**Table 4:** Urinalysis findings five days after hospital admission Urinalysis results obtained five days after hospital admission, demonstrating positive nitrites, abundant leukocytes, abundant bacteria, and persistent hematuria, consistent with progression of urinary inflammatory findings. Patient values are presented alongside the corresponding reference ranges and units.

Urinalysis	Patient’s value	Reference range	Units	
Color	Brown	Pale yellow to amber	N/A	
Appearance	Cloudy	Clear - Transparent	N/A	
Density	1.02	1.005 - 1.030	N/A	
pH	5	4.5 - 8.0	N/A	
Protein	25	Negative	mg/dL	
Glucose	Normal	Negative	mg/dL	
Ketones	5	Negative	mg/dL	
Hemoglobin	250	Negative	Ery/uI	
Nitrites	Positive	Negative	N/A	
Leukocytes per high-power field	Abundant	0 - 5	/HPF	
Erythrocytes per high-power field	Abundant	0 - 3	/HPF	
Bacterium	Abundant	Absent to rare	/HPF	

A lower midline infraumbilical approach was selected to provide adequate exposure of the hernia defect and allow controlled visualization of the herniated bladder during reduction, facilitating bladder preservation. A right direct inguinal hernia defect measuring approximately 4 cm in diameter was identified, containing a component of the urinary bladder and structures of the spermatic cord (Figure 4). The herniated bladder was reduced using external maneuvers, and the defect was repaired by primary tissue herniorrhaphy with preservation of the inguinal canal structures and confirmation of complete closure. Prosthetic mesh was not used given the clinically suspected urinary infection and the feasibility of primary tissue repair. Right testicular hypoplasia was also noted intraoperatively; its clinical significance and relationship to the chronic hernia remain uncertain.

**Figure 4:**
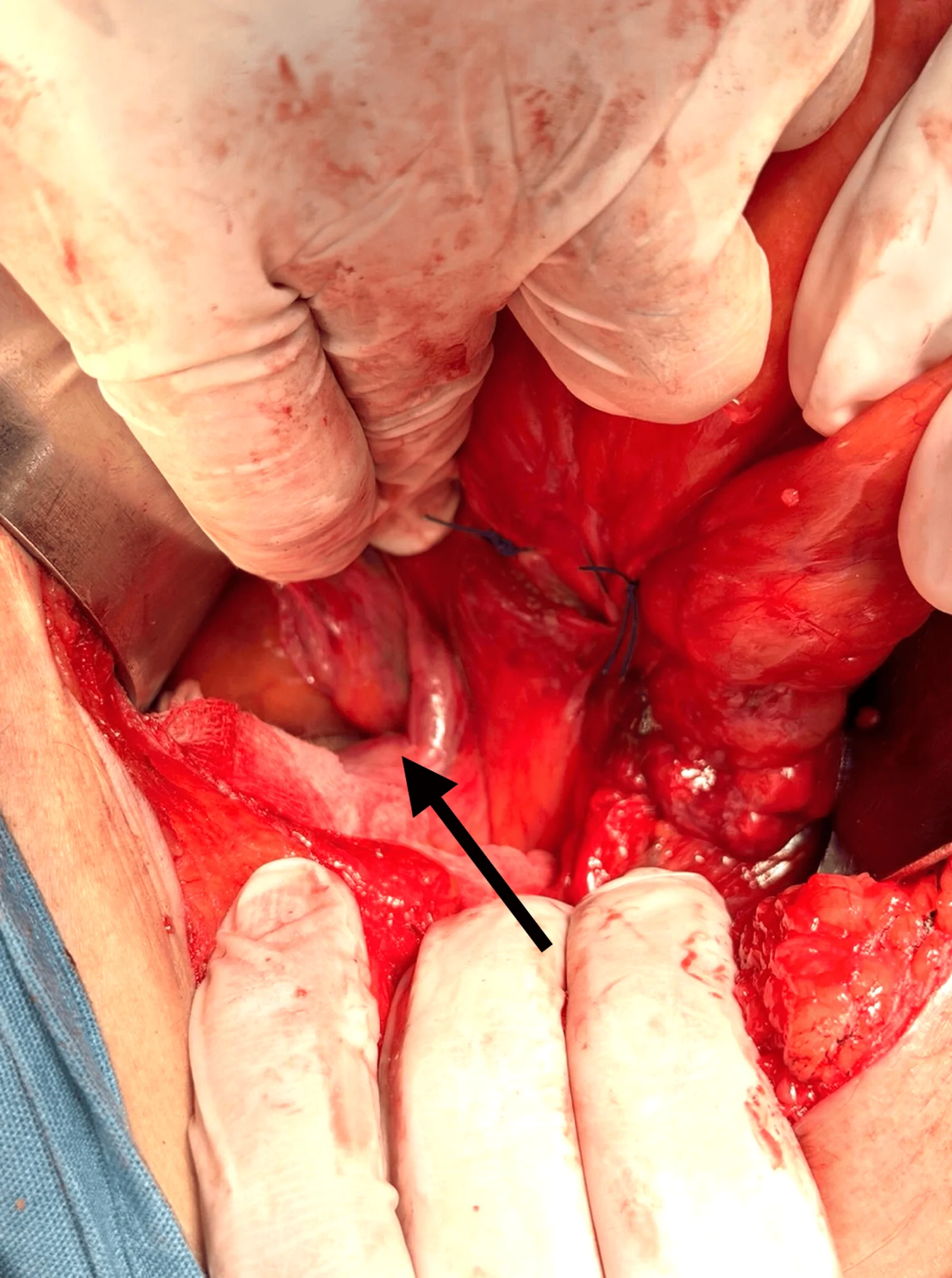
Intraoperative posterior view of the inguinal defect during primary repair Intraoperative photograph showing the right inguinal hernia defect during primary tissue repair, with partial closure using sutures.

Postoperatively, the patient initially continued to experience febrile episodes and required escalation of antimicrobial therapy. His inflammatory response subsequently improved, with C-reactive protein decreasing from 276 to 63.7 mg/L (reference range: 0-5 mg/L) and normalization of the white blood cell count. The final urine culture showed no growth. Renal function improved, with serum creatinine decreasing to 1.2 mg/dL two days after surgery and subsequently to 0.8 mg/dL. The surgical wound remained clean, without evidence of surgical-site infection or other postoperative complications. The patient was discharged in stable condition after improvement in the urinary and systemic inflammatory findings.

At two-month follow-up, there was no clinical evidence of hernia recurrence. The patient remained under multidisciplinary follow-up with the Internal Medicine, Urology, and Geriatrics services.

## Discussion

Inguinal herniation of the urinary bladder is an uncommon manifestation of inguinal hernia, accounting for less than 4% of cases and occurring predominantly in older men. It is frequently associated with lower urinary tract symptoms and may remain clinically unrecognized when bladder involvement is not suspected. A systematic review of 64 patients reported lower urinary tract symptoms in approximately half of cases, while manual compression of the inguinoscrotal mass during voiding was less frequently described [1,2].

Mery’s sign may provide an important clinical clue to bladder involvement. It is characterized by the need to manually compress the inguinal or scrotal mass to facilitate bladder emptying and may be particularly suggestive in patients with chronic inguinoscrotal masses and two-stage voiding [2-4]. In the present case, compression of the scrotal mass resulted in increased urinary drainage through the catheter, supporting the clinical suspicion of bladder herniation. Recognition of this finding may facilitate preoperative diagnosis.

Preoperative identification is clinically relevant because unrecognized bladder involvement may increase the risk of bladder injury during hernia repair. CT is particularly useful for confirming bladder herniation, defining its extent, and evaluating associated urinary tract abnormalities [1,5,6]. In our patient, CT confirmed bladder involvement and provided important anatomical information for surgical planning.

Bladder-containing inguinal hernias may be associated with urinary obstruction, hydronephrosis, renal dysfunction, and urinary tract infection, with severe cases progressing to systemic infection [7-11]. These complications are thought to result primarily from impaired bladder emptying, mechanical distortion, and urinary stasis. In the present case, the patient had progressive lower urinary tract symptoms, hematuria, and renal dysfunction, followed during hospitalization by fever, leukocytosis, flank pain, and worsening urinary and inflammatory findings despite initial antimicrobial therapy. These changes prompted reassessment of the initial conservative approach and ultimately led to definitive surgical intervention.

The optimal timing of repair in patients with bladder-containing inguinal hernias and suspected urinary or systemic inflammatory complications is not well established. Available evidence is largely derived from case reports and small series describing urinary decompression, antimicrobial therapy, clinical stabilization, and subsequent hernia repair [8-10,12]. In our patient, surgery was initially deferred to allow clinical optimization; however, persistent and worsening inflammatory findings despite initial medical management led to definitive repair five days after admission. This sequence should be interpreted as an individualized management decision rather than an established treatment algorithm.

Surgical treatment generally consists of reduction of the herniated bladder followed by repair of the hernia defect. Bladder resection is generally reserved for selected circumstances, including bladder wall necrosis, a true diverticulum, severe constriction of the hernia neck, or associated bladder neoplasia [1,2]. Open, laparoscopic, and robotic approaches have all been described [1,3,5,6,10-12]. In the present case, a lower midline infraumbilical approach was selected to provide adequate exposure of the hernia defect and allow controlled visualization during bladder reduction. The bladder was successfully reduced, and a right direct inguinal defect measuring approximately 4 cm was repaired by primary tissue herniorrhaphy without prosthetic material.

The decision not to use synthetic mesh in this patient should be interpreted as an individualized surgical decision rather than a general recommendation. Mesh-based repairs have been successfully reported in patients with bladder-containing inguinal hernias, including cases associated with urinary infection, obstruction, or renal dysfunction [11,12]. Therefore, suspected urinary infection or inflammatory findings do not necessarily preclude mesh repair. In our patient, the combination of clinically suspected urinary infection, systemic inflammatory findings, and the feasibility of primary closure of the approximately 4 cm defect led the surgical team to favor primary tissue repair.

Evidence specifically addressing prosthetic mesh use in bladder-containing inguinal hernias with suspected urinary infection remains limited. Consequently, the decision regarding prosthetic material should be individualized according to the patient's clinical condition, degree of contamination or inflammation, tissue quality, anatomical characteristics, and feasibility of primary closure. The present case does not establish that synthetic mesh should be routinely avoided in patients with suspected urinary infection.

The favorable postoperative course demonstrates that primary repair may be feasible in selected patients when adequate tissue approximation can be achieved. Following surgery and antimicrobial escalation, renal function recovered and inflammatory parameters decreased, and the patient was discharged without surgical complications. No early recurrence was observed at two-month follow-up. However, the final urine culture showed no growth, making the diagnosis of urinary tract infection less definitive and potentially reflecting prior antimicrobial exposure. In addition, the short follow-up period limits any meaningful assessment of long-term recurrence or late complications.

The principal clinical lesson is that bladder involvement should be considered in older men with inguinoscrotal hernias accompanied by lower urinary tract symptoms, particularly two-stage voiding or the need for manual compression during urination. Mery’s sign may raise clinical suspicion, while CT can establish the diagnosis and facilitate surgical planning. When urinary or systemic inflammatory findings are present, the timing and technique of repair should be individualized according to the clinical response, anatomical findings, bladder viability, and feasibility of repair. Primary tissue repair without prosthetic mesh may be feasible in selected cases; however, this single case cannot establish a preferred surgical strategy or support routine avoidance of mesh.

Limitations

This report has several limitations. First, as a single case report, the findings cannot be generalized to all patients with bladder-containing inguinoscrotal hernias or used to establish an optimal timing or surgical technique. Second, the two-month follow-up period was too short to meaningfully assess long-term hernia recurrence or other late complications. Third, although the patient developed clinical and laboratory findings suggestive of a urinary tract infection during hospitalization, the final urine culture showed no growth, making the diagnosis of urinary tract infection less definitive and potentially reflecting prior antimicrobial exposure. Finally, the decision to perform primary tissue repair without prosthetic mesh was based on the patient's clinical condition, the presence of systemic inflammatory findings, and the feasibility of primary closure; therefore, this approach should not be interpreted as evidence that mesh repair is contraindicated in patients with suspected urinary tract infection.

## Conclusions

Inguinoscrotal herniation of the urinary bladder is an uncommon condition that may be overlooked when urinary symptoms are not recognized as manifestations of bladder involvement. Mery's sign should increase clinical suspicion in older men with inguinal or scrotal masses and lower urinary tract symptoms, while CT is valuable for confirming bladder involvement and facilitating surgical planning. In patients with suspected urinary infection or systemic inflammatory findings, management should be individualized according to the patient's clinical condition and operative findings. This case highlights the importance of preoperative recognition, close clinical reassessment, and individualized surgical decision-making in patients with bladder-containing inguinoscrotal hernias; however, a single case cannot establish a preferred repair strategy or support routine avoidance of prosthetic mesh.

## References

[1] Branchu B, Renard Y, Larre S, Leon P (2018). Diagnosis and treatment of inguinal hernia of the bladder: a systematic review of the past 10 years. Turk J Urol.

[2] Kraft KH, Sweeney S, Fink AS, Ritenour CW, Issa MM (2008). Inguinoscrotal bladder hernias: report of a series and review of the literature. Can Urol Assoc J.

[3] Peñacoba LM, Aurusa JO, Martín ME, Perea JG, Rentería JB (2015). Bladder hernia and Mery's sign: a case report (Article in Spanish). Rev Hispanoam Hernia.

[4] Elkbuli A, Narvel RI, McKenney M, Boneva D (2019). Inguinal bladder hernia: a case report and literature review. Int J Surg Case Rep.

[5] Gonzalez-Urquijo M, Mayagoitia-Gonzalez JC, Rodarte-Shade M, Bermea-Mendoza JH, Gil-Galindo G (2019). Large inguinal bladder hernias: can a preoperative diagnosis be made?. Hernia.

[6] Papatheofani V, Beaumont K, Nuessler NC (2020). Inguinal hernia with complete urinary bladder herniation: a case report and review of the literature. J Surg Case Rep.

[7] Bisharat M, O'Donnell ME, Thompson T, MacKenzie N, Kirkpatrick D, Spence RA, Lee J (2009). Complications of inguinoscrotal bladder hernias: a case series. Hernia.

[8] Cullen GD, Singh P, Gregoire JR (2021). Inguinoscrotal herniation of the bladder: a rare case of obstructive uropathy. Urol Case Rep.

[9] Wagner AA, Arcand P, Bamberger MH Acute renal failure resulting from huge inguinal bladder hernia. Urology.2004.

[10] Safavy S, Mitsinikos E, Tropea B, Chang A, Patel H (2018). Obstructive uropathy and sepsis caused by an inguinoscrotal bladder hernia: a case report. Perm J.

[11] Karanikas M, Kofina K, Foutzitzi S, Deftereos S, Effraemidou E, Lyratzopoulos N, Polychronidis A (2020). Urinary bladder-containing incarcerated inguinoscrotal hernia: a case report. J Surg Case Rep.

[12] Chang HH, Wen SC (2021). Extraperitoneal laparoscopic repair of huge inguinoscrotal bladder hernia: a case report and literature review. Urol Case Rep.

